# Two-Step Magnetization Reversal FORC Fingerprint of Coupled Bi-Segmented Ni/Co Magnetic Nanowire Arrays

**DOI:** 10.3390/nano8070548

**Published:** 2018-07-19

**Authors:** Javier García Fernández, Víctor Vega Martínez, Andy Thomas, Víctor Manuel de la Prida Pidal, Kornelius Nielsch

**Affiliations:** 1 Leibniz Institute for Solid State and Materials Research (IFW) Dresden, Helmholtzstraße 20, 01069 Dresden, Germany; a.thomas@ifw-dresden.de (A.T.); k.nielsch@ifw-dresden.de (K.N.); 2 Departamento de Física, Universidad de Oviedo, C/Federico Garcia Lorca 18, 33007 Oviedo, Asturias, Spain; vegavictor@uniovi.es (V.V.M.); vmpp@uniovi.es (V.M.d.l.P.P.); 3 Laboratorio Membranas Nanoporosas, Servicios Científico-Técnicos, Universidad de Oviedo, Campus El Cristo s/n, 33006 Oviedo, Asturias, Spain

**Keywords:** nanowires, electrochemical deposition, First Order Reversal Curves, two-step magnetization reversal

## Abstract

First Order Reversal Curve (FORC) analysis has been established as an appropriate method to investigate the magnetic interactions among complex ferromagnetic nanostructures. In this work, the magnetization reversal mechanism of bi-segmented nanowires composed by long Co and Ni segments contacted at one side was investigated, as a model system to identify and understand the FORC fingerprint of a two-step magnetization reversal process. The resulting hysteresis loop of the bi-segmented nanowire array exhibits a completely different magnetic behavior than the one expected for the magnetization reversal process corresponding to each respective Co and Ni nanowire arrays, individually. Based on the FORC analysis, two possible magnetization reversal processes can be distinguished as a consequence of the ferromagnetic coupling at the interface between the Ni and Co segments. Depending on the relative difference between the magnetization switching fields of each segment, the softer magnetic phase induces the switching of the harder one through the injection and propagation of a magnetic domain wall when both switching fields are comparable. On the other hand, if the switching fields values differ enough, the antiparallel magnetic configuration of nanowires is also possible but energetically unfavorable, thus resulting in an unstable magnetic configuration. Making use of the different temperature dependence of the magnetic properties for each nanowire segment with different composition, one of the two types of magnetization reversal is favored, as demonstrated by FORC analyses.

## 1. Introduction

The continuous growth of the nanotechnology makes the understanding of nature at such low scale necessary to develop more advanced devices. Generally, high yielding fabrication processes of nanomaterials do not allow to produce perfectly identical nanostructures, whose functional properties differ slightly one from another. Then, it is a challenge to measure and define the main parameters that represent the population of the fabricated nanostructures. In this context, self-ordered porous Anodic Aluminum Oxide membranes (AAOs) when used as templates, have been established as a large scale, suitable and low cost fabrication technique of a wide range of nanomaterials such as antidot thin films, nanotubes and nanowires, among others [1,2,3,4]. When the pores of the AAO membranes are filled with a metallic material by means of electrodeposition techniques, the deposited material reproduces the pore shape, thus forming a self-ordered nanowire array [5,6]. Among the applications in other research fields such as photonics, electronics and sensing devices, ferromagnetic nanowires are also interesting for high density magnetic data storage and spintronics [7]. The latter application has been a subject of extensive study and requires the control of magnetic domain walls and magnetization reversal processes of the nanomaterial. With this purpose, heterogeneous ferromagnetic nanowires are being fabricated by modulating their shape, changing their diameter, and/or their composition, performing multi-segments and multi-layers [4,8,9,10,11,12]. Nevertheless, due to the low dimensions of the nanowires, their magnetic characterization is frequently performed when nanowires remain embedded into the pores of the alumina membrane, in order to have enough signal when many nanowires contribute to the total magnetization of the sample. However, when the nanowires are still arranged in the alumina template, the magnetostatic interactions among the nanowires become important due to the short inter-nanowire distances [13,14]. Consequently, it is still a challenge to extrapolate individual magnetic behaviors of complex nanowires systems from the macroscopic magnetic characterization of the whole nanowire array. 

The FORC analysis is in general a method to evaluate a hysteretic behavior whose detailed measuring principles and interpretations on different particle systems can be found in the literature [15]. When applied to magnetism, the main idea behind the method is to consider that the major hysteresis loops are a superposition of individual and, in principle, indivisible elements of hysteresis called as “hysterons”. Starting from positive saturation, the applied magnetic field is reduced to a certain value and increased again back to saturation recording the first order reversal curve itself. Then, this process is repeated for several reversal fields (Hr) to cover the whole descendant branch of the hysteresis loop. Finally, the FORC distribution is calculated from the mixed derivative of the magnetization with respect to the reversal (Hr) and applied (H) fields, as shown in Equation (1), thus evaluating the change on the magnetic susceptibility at different first order reversal curves.

(1) $$\rho_{\mathrm{FORC}}\left(H,H_{r}\right)=-\frac{1}{2}\frac{\partial^{2}m}{\partial H_{r}\partial H}$$

However, it is worth mentioning that, due to time considerations as well as technical limitations, in a FORC experiment, a reversal performed nanowire by nanowire (hysteron by hysteron) is rather difficult. In turn, the magnetic field step will reverse a group of hysterons, so the resulting apparent hysteron will be composed of a group of hysteretic elements. This fact will make the FORC diagram to be formed of “bigger” pixels, although, if the field step is fine enough, it can still reproduce a holistic view of the system. The aim of this work was to identify the FORC fingerprint of a two-step magnetization reversal process proposing a model to validate the experimental FORC analysis performed in a type system. For this purpose, a bi-segmented Ni/Co nanowire array composed by a 20 µm Ni long segment followed by 20 µm Co one was studied. The effect of the exchange coupling and/or magnetostatic interactions at the interface layer over the magnetic properties when both composition segments are in contact was investigated. The limitations of performing the magnetic characterization of a single ferromagnetic nanowire make the understanding of the magnetic behaviors of these complex systems difficult. However, First Order Reversal Curve (FORC) analysis provides valuable information concerning the intrinsic properties of the nanowires as well as the magnetic interactions among them [16,17,18].

## 2. Materials and Methods

### 2.1. Samples Fabrication and Characterization

**Figure 1:**
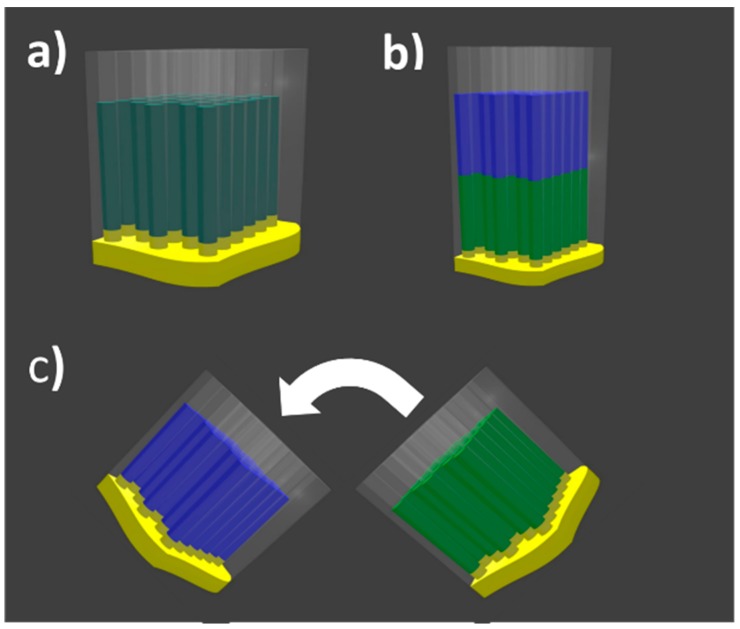
Schematic drawings of the different samples: (**a**) single element Ni or Co nanowire arrays; (**b**) coupled Ni/Co bi-segmented nanowire array; and (**c**) decoupled Ni/Co nanowire array where the arrow represents the direction of the arrays stacking. In all figures, the Ni and Co segments are green and blue respectively, while the Au bottom contact is yellow.

AAO membranes were fabricated by means of the well-known two step anodization process, whose general details can be found elsewhere [19]. Particularly, for this study, the AAO membranes were anodized in 0.3 M oxalic acid at 3 °C leading to a pore diameter of around 35 nm and distance among adjacent pores of 105 nm. The total anodization time was chosen so that the thickness of the AAO membranes would be 50 µm in all cases. Further, the remaining aluminum substrate was removed from the bottom side of the alumina membrane with a solution of 36 g/L of CuCl_2_·2H_2_O + 500 mL/L of HCl at 37%. The barrier layer of the AAO membranes that occludes the bottom part of the pores was removed by wet chemical etching in 5%wt phosphoric acid. After opening the barrier layer, the mean pore diameter was found to be approximately 42 nm, while the interpore distance of 105 nm remained unaltered during the etching process. As the electrodeposition of metallic material requires an electrical contact, a thin Au film was sputtered on one side of the alumina membrane. This process was followed by Au electroplating to perform a homogeneous contact occluding completely the channels at this side of the AAO membranes. To compare the effect of the coupling between the two ferromagnetic segments with different composition, arrays of same dimensions but only filled with Ni or Co segments were fabricated as a reference of each nanowires composition (Figure 1a). The electrodeposition of Co and Ni nanowires was carried out by means of the Watts type electrolyte with fixed pH of 4.3: 300 g/L NiSO_4_·6H_2_O + 45 g/L NiCl_2_·6H_2_O + 45 g/L H_3_BO_3_ and 300 g/L CoSO_4_·7H_2_O + 45 g/L CoCl_2_·6H_2_O + 45 g/L H_3_BO_3_, respectively. The temperature of the electrolytes was kept at 45 °C during the electrodeposition to avoid boric acid precipitation. Co and Ni nanowires were potentiostatically electrodeposited at fixed −1 V and −1.2 V vs. Ag/AgCl reference electrode, respectively, for 10 min in both cases. The bi-segmented Ni/Co nanowire array represented in Figure 1b, hereafter referred to as coupled Ni/Co sample, was prepared by a first electrodeposition of pure Ni long segment and a subsequent electrodeposition of pure Co long segment by sequentially using the two different electrolytes above mentioned. Although the single segment samples provide information regarding the intrinsic magnetization reversal behavior of the single element array, two pieces of Co and Ni nanowire arrays were also stacked and measured together to reproduce a non-interacting bimagnetic system (decoupled Ni/Co sample from now on), where the Ni and Co segments were not directly in contact and far from each other (Figure 1c). Even if the hysteresis loop of the decoupled Ni/Co sample was expected to show a trivial superposition of the single element Co and Ni arrays, the FORC diagram of this system could show unexpected contributions due to the presence of the two different magnetic materials, which should be considered in the interpretation of the coupled Ni/Co FORC results.

Finally, morphological and compositional characterization have been performed by means of a Scanning Electron Microscope (SEM, JEOL 6100 LV, JEOL, Akishima, Tokyo, Japan) equipped with an Energy Dispersive X-ray microanalysis system (EDX, Inca Energy 200, Oxford Instruments, Abingdon, UK). The length of the Co and Ni segments after 10 min of electrodeposition is around 16 μm and 20 μm in each case, respectively, thus the high aspect ratio of the nanowires is assured. On the other hand, magnetic hysteresis loops carried out along parallel and perpendicular directions to nanowires long axis, together with FORC measurements along the parallel direction and at different temperatures, have been performed in a Vibrating Sample Magnetometer (VSM-Versalab, Quantum Design, San Diego, CA, USA) under a magnetic field up to ±3T and in the temperature range between 50 K and 400 K. 

### 2.2. Statistical Approach of Modelling FORC Simulations

The FORC method has been widely employed for the evaluation of individual properties of ferromagnetic nanoentities as extracted from macroscopic measurements such as in the case of magnetic nanowire arrays [17,18,20]. This method is based on the assumption that, in this kind of systems, the major magnetic hysteresis loop corresponds to the superposition of several magnetization reversal processes that may be attributed to the contribution of each nanowire into the array. Then, the reversal of the magnetization for each nanowire can be considered as an indivisible process of hysteresis called *hysteron*. This entity can be defined by its two intrinsic switching fields $H_{b}$ and $H_{r}$. The first one corresponds to the applied magnetic field necessary to saturate the hysteron along the positive direction of magnetization, while *H_r_* is the field necessary to saturate the hysteron back to negative saturation. Through the change of coordinates $H_{c}=\left(H_{b}-H_{r}\right)/2$ and $H_{u}=-\left(H_{b}+H_{r}\right)/2$, it is possible to achieve valuable information concerning the intrinsic magnetic anisotropies and the magnetostatic interactions among the nanowires, respectively. Taking into account that, from the experimental point of view, not all the nanowires are identical in size and/or composition, the system can be described with a distribution of *hysterons* whose magnetic properties differ slightly from the mean ones. In this case, a Gaussian type distribution of *hysterons*, as in Equation (2), has been chosen through defining its mean switching field $\langle H_{SW}\rangle$ and with its standard deviation (σ):

(2) $$G\left(H,\langle H_{SW}\rangle \right)=\frac{1}{\sigma \sqrt{2\pi }}e^{-\frac{{\left(H-\langle H_{SW}\rangle \right)}^{2}}{2\sigma^{2}}}$$

From previous equation, and modifying the expression to obtain normalized values, the magnetization can be obtained by a direct integration of the *hysterons* distribution:

(3) $$m\left(H_{b},H_{r}\right)=\left[m_{Hr}+1+\mathrm{erf}\left(\frac{H_{b}-\langle H_{SW}\rangle }{\sqrt{2\sigma^{2}}}\right)\right]\cdot \theta \left(-H_{r}-H_{b}\right)+\theta \left(H_{b}+H_{r}\right)$$

where $m_{Hr}$ denotes the value of the magnetization at a certain reversal field $H_{r}$. This value is obtained from the integration of the negative hysteron distribution centered at $-\langle H_{SW}\rangle$, i.e., $-G\left(H,-\langle H_{SW}\rangle \right)$. Furthermore, θ is the Heaviside function that takes the value 1 when its argument is positive and 0 otherwise. Then, the Heaviside function is important to take into account when the magnetization in Equation (3) reaches its saturation state. Finally, the switching process of one of the nanowires in the array is able to modify the overall magnetic field felt by its neighbors due to the spatial proximity among them. If the switching process is supposed to be homogeneous enough along the whole array, its effect is to produce an interaction magnetic field, called mean field interaction (MFI), pointing along the opposite direction with respect to the direction of the magnetization, which can be seen as a demagnetizing field [21,22,23]. In other words, the effective magnetic field that is felt by the nanowires in the array is reduced by a factor that is proportional to, among others, the distance among nanowires and their saturation magnetization [24]. This contribution can be added into the above equations through a renormalization of the magnetic field, distinguishing between both the applied and effective magnetic fields, $H_{app}$ and $H_{eff}$, respectively, [25,26,27,28] as Equation (4).

(4) $$H_{eff}=H_{app}+\alpha m$$

where the negative interaction of magnetostatic origin among nanowires can be represented by α<0, and denotes the strength of the interaction field at saturation (m=1). 

**Figure 2:**
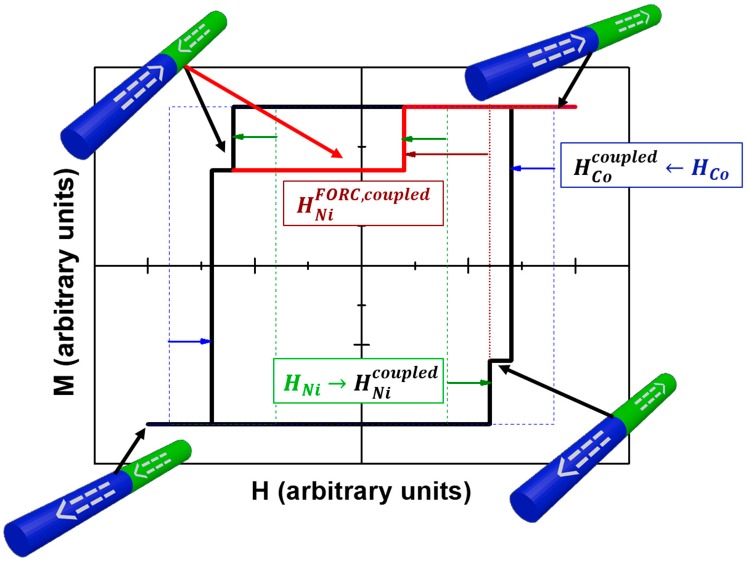
Schematic representation of the two-step magnetization reversal hysteresis loop of a bisegmented Ni(green)/Co(blue) nanowire. Dashed lines denote the intrinsic hysteresis loops for Ni and Co segments. The arrows indicate the change on the switching fields with respect their intrinsic values due to the magnetic coupling between the Co and Ni segments. Red line indicates the expected behavior of a first order reversal curve starting from the antiparallel configuration.

Finally, to reproduce the magnetic behavior of the coupled Ni/Co bisegmented nanowire array, the contribution of exchange coupled segments must be included. The schematic representation of such magnetic behavior is shown in Figure 2. Let us consider the case of a single bisegmented nanowire composed of a Ni and Co segments with their respective intrinsic switching fields $H_{Ni}$ and $H_{Co}$ when $H_{Ni}<H_{Co}$. Starting from positive saturation and then decreasing the applied magnetic field, the first switching event to occur would come from the Ni segment. However, as it is coupled with the Co segment whose magnetization is still pointing along the positive direction, the effective switching field of the coupled Ni segment is slightly increased $H_{Ni}^{coupled}>H_{Ni}$. Furthermore, it is reasonable to think that further decreasing the magnetic field, the switching of Ni segment will induce the Co segment to switch earlier than its intrinsic value $H_{Co}^{coupled}<H_{Co}$. In terms of FORC, starting the curve from the antiparallel configuration (red line in Figure 2), the Ni segment is forced to switch before its intrinsic switching field due to the magnetic interaction with the Co one so, $H_{Ni}^{FORC,coupled}<H_{Ni}<H_{Ni}^{coupled}$. 

## 3. Results and Discussion

### 3.1. Ni and Co Nanowire Arrays

**Figure 3:**
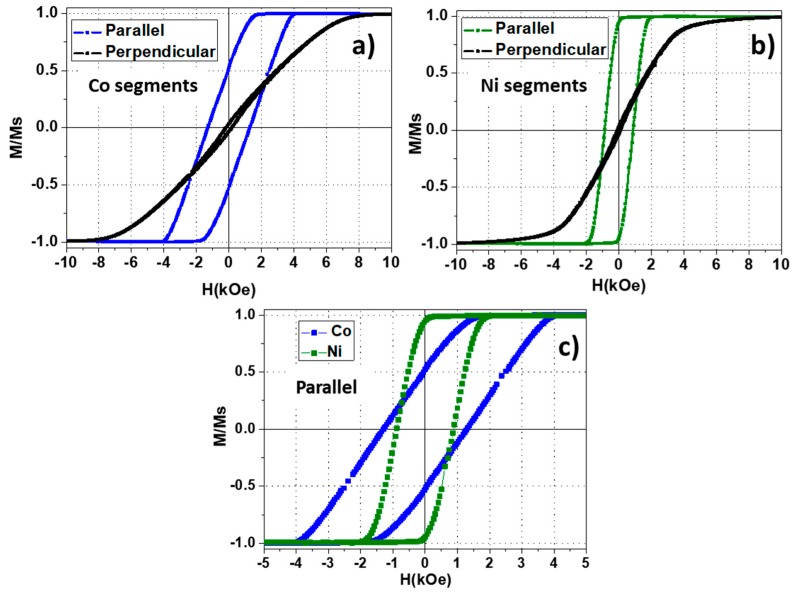
Room temperature hysteresis loops measured along the parallel and perpendicular (black) direction with respect to the nanowire long axis in (**a**) Co (blue) and (**b**) Ni (green) segment arrays. The comparison between the parallel hysteresis loops of Co and Ni segment arrays is shown in (**c**).

To understand the magnetic behavior of the coupled Ni/Co bisegmented nanowires sample, the intrinsic properties of the single element arrays must be magnetically characterized in detail first. In Figure 3a,b, the room temperature hysteresis loops measured along both the parallel and perpendicular directions with respect the nanowire length axis are shown. First, in both cases, one can observe a well-defined uniaxial magnetic anisotropy with its easy magnetization axis lying parallel to the nanowires long axis. Moreover, by comparing each parallel hysteresis loops (Figure 3c), Co segments exhibit larger coercive field ($H_{c}^{Co}=1296\;Oe$) than Ni ones ($H_{c}^{Ni}=884\;Oe$). The fact that Co hysteresis loop is more tilted along the magnetic field axis, while maintaining its width, evidences that such segments are submitted to stronger magnetostatic interactions, associated to its stronger saturation magnetization value.

**Figure 4:**
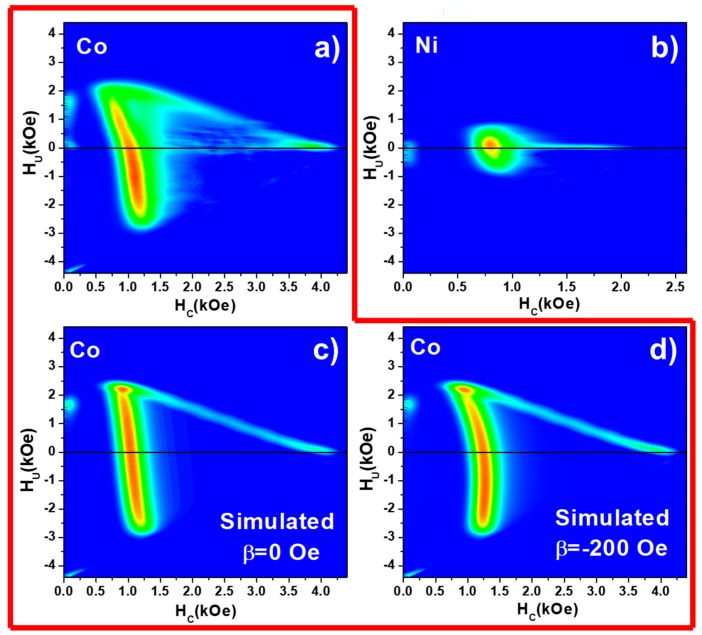
Experimental FORC diagrams of Co (**a**) and Ni (**b**) segment arrays measured along the nanowires axis. Simulated FORC diagrams for Co nanowire array with fixed experimental values of SFD and α, for β = 0 Oe (**c**) and β = −200 Oe (**d**).

To extract the intrinsic switching field distribution (SFD) of the Co and Ni nanowire arrays and evaluate the magnetostatic interaction among them, FORC analysis was performed on these samples. The parallel FORC diagrams of both samples are presented in Figure 4a,b. First, in the case of the Co nanowire array, the FORC distribution shows two clear branches, which are typically present in systems where the switching field distribution of hysterons and antiparallel magnetostatic interactions coexist. These two branches of the FORC diagram were referred to as “wishbone” shape by Pike et al., who demonstrated experimentally and theoretically its origin and settled a guide to extract the relevant information from this kind of FORC diagrams [29]. However, it results non-trivial the bending of the distribution to lower *H_C_* values when *H_u_* is negative. Such behavior is not ascribed to an asymmetry of the SFD, but instead to an inhomogeneity of the mean field interaction. As discussed in Section 2.2, under the MFI theory, the magnetostatic interaction among nanowires in the array is proportional to the magnetization. This means that, at the coercive field value where the net magnetization is negligible, the strength of magnetostatic interaction should also become zero. However, the characteristic arrangement of nanowires in case of AAO templates can play an important role on how the nanowires interact near the coercive field of the system. This inhomogeneity can be taken into account by adding a second order term to the MFI as follows [24,30]:

(5) $$H_{eff}=H_{app}+\alpha m+\beta \left(1-m^{2}\right)$$

In Figure 4c,d, the effect of the *β* parameter on simulated FORC distributions can be observed. The major effect is to bend the upper part of the vertical branch towards lower values of H_C_ and to displace the main peak to negative values of the interaction field. The effect of *β* on $H_{eff}$ at the coercive field (m=0 in Equation (5)) points out that the coercive field extracted from the major hysteresis loop does not correspond to the maximum of the SFD in the FORC distribution (see Table 1), showing the differences between the coercive field of the array and the switching field of the nanowires. 

Moreover, the experimental diagram shown by the Ni nanowire array corresponds to the so-called “T-shape”. Under the Preisach model, such diagram has been wrongly proposed in the past to be a consequence of a second magnetically harder phase present in the sample which, in addition, would not be interacting. However, the existence of such magnetic phase with different switching fields is hardly understood (up to 2000 Oe), while the main distribution is around 900 Oe. Furthermore, both crystalline structure and morphology of the electrodeposited Ni nanowires are very homogeneous, which would reduce the probability to find different magnetic phases or nanowires having such a difference in their switching fields [31]. That shape of the FORC diagram of Ni nanowire array was explained in detail by Dobrota et al. [32], concluding that it is a consequence of a nonlinear dependence of the interaction field with respect to the normalized magnetization, i.e., the value of the interaction among wires depends on the magnetization state of the sample [33,34]. Nevertheless, by comparing the FORC diagrams of Ni and Co nanowire arrays, the stronger magnetostatic interaction among Co nanowires is evidenced again, as pointed out by the higher value of α in Table 1. As a summary of the magnetic behavior of the single element nanowire arrays (Table 1), Co segments are magnetically harder than the Ni ones, with stronger magnetostatic interactions due to the higher saturation magnetization of Co (162.7 emu/g) with respect Ni (57.5 emu/g) [35].

### 3.2. Coupled and Decoupled Ni/Co Bi-Magnetic Systems

**Figure 5:**
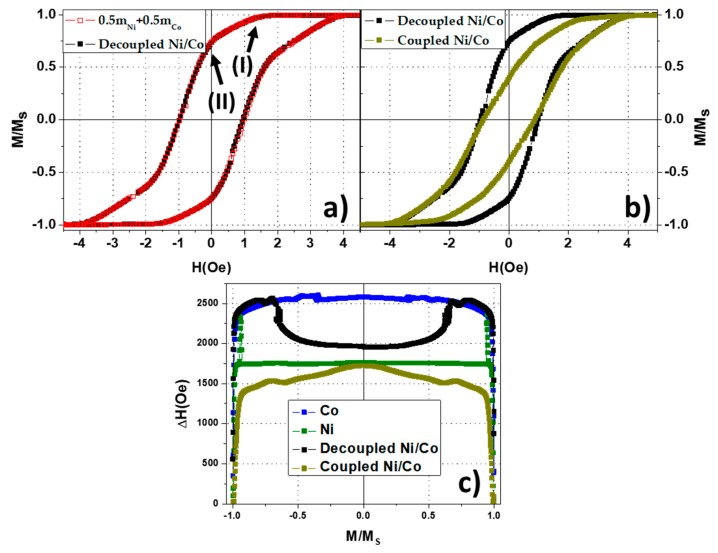
(**a**) Numerical superposition of the Co and Ni room temperature hysteresis loops (red open symbols) compared to the decoupled Ni/Co hysteresis loop (black). (**b**) Comparison between the decoupled (black) and coupled (yellow) hysteresis loops. (**c**) Comparison of the hysteresis shape plots (width of hysteresis loops at different values of magnetization.

As presented in the previous sections, the easy magnetization axis of the nanowire arrays lies parallel to the nanowires long axis. Therefore, the following studies focused on that direction along which the hysteretic magnetization switching processes take place. In Figure 5a, the major hysteresis loops of Ni and Co nanowire arrays are numerically superimposed in a proportion 1:1 and are compared with the non-interacting segments of the decoupled Ni/Co nanowire array. As expected, the resulting magnetic behavior of the decoupled Ni/Co sample corresponds to the superposition of the independent hysteresis loops of Ni and Co nanowires, reproducing a system where there is no magnetic interaction between segments of each different element. Paying attention to the descendant branches of the hysteresis loops, it is possible to distinguish the contribution of each type of segment. First, as the magnetic field is reduced in the decoupled sample, the magnetization drops down at the same value as the Co nanowire array does it (Point I). Further decrease of the magnetic field provokes a second drop of the magnetization corresponding to the magnetization switching of Ni segments (Point II). The hysteresis loop of the coupled Ni/Co sample is compared with the hysteresis loop of the decoupled sample in the Figure 5b, where the differences are clearly evidenced. Figure 5c is another representation of the differences among the above samples emphasizing on their hysteresis. Such representation consists on the plot of the hysteresis loop width at different values of the reduced magnetization. In the case of the decoupled Ni/Co sample, the hysteresis of each composition is clearly evidenced, while, in the case of coupled Ni/Co nanowire array, such differences are suppressed and the overall magnetic hysteresis is close to that shown by the Ni nanowire array. As a first approximation, the hysteresis and the tilt of the coupled Ni/Co hysteresis loop resembles to the equivalent system with the switching field of a Ni nanowire array with the MFI strength presented by the Co one. 

**Figure 6:**
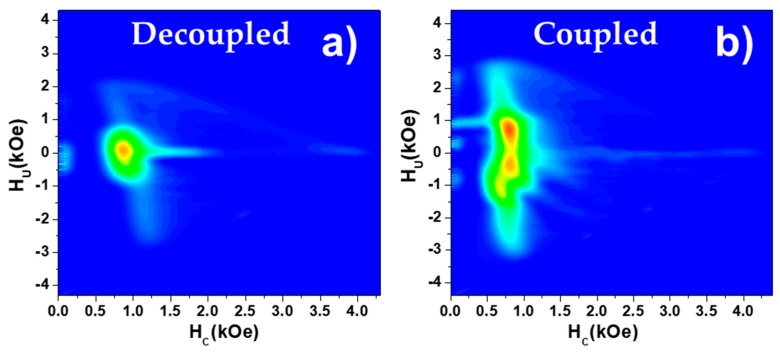
Parallel FORC diagrams, measured along the easy magnetization axis, in decoupled (**a**) and coupled (**b**) Ni/Co nanowire arrays.

To obtain more detailed magnetic information of such magnetostatically interacting nanowire arrays, FORC analyses were also compared. The FORC diagram of a decoupled Ni/Co nanowire array corresponds again to the superposition of the individual FORC diagrams of Ni and Co nanowire arrays, as shown in Figure 6a. However, the differences between the coupled Ni/Co FORC diagram and the previous one point out that some additional effect must be taken into account. As the unique difference between both samples lies on the fact that the nanowires segments are in contact, it is reasonable to think that exchange coupling or magnetostatic interactions at the interface between the two nanowire segments is the responsible of such a complex FORC distribution. As a first approximation, one can consider two main mechanisms for the magnetization reversal process of one of the Ni/Co bi-magnetic nanowire. On the one hand, if the switching fields of both segments are close enough, the magnetization reversal of the whole nanowire could be carried out in a single step through injection and propagation of a domain wall from the magnetically softer to the harder segment to avoid an unstable antiparallel configuration of the magnetic moments. This case would result in a one-step magnetization reversal of the Ni/Co nanowire with an effective switching field dominated by the magnetically softer phase, i.e., Ni segment in this case, whilst the mean field interaction would be increased by the segment having higher saturation magnetization (Co segment in this case) [36]. On the other hand, if the switching fields of each kind of segments are different enough, the magnetization reversal would not be carried out in a single step, which would be reflected with two differentiated distributions in the FORC, as observed in Figure 6b.

**Figure 7:**
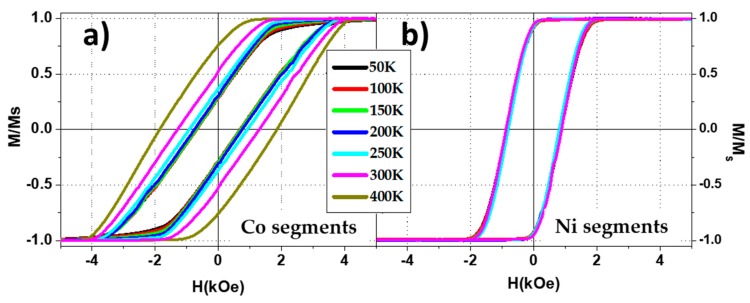
Parallel hysteresis loops measured at different temperatures ranging from 50 K to 400 K for Co (**a**) and Ni (**b**) nanowire arrays.

Since the difference between the switching fields of Ni and Co segments appears to be the main parameter that controls which kind of magnetization reversal process is promoted, the different temperature dependence of the switching fields in both cases can be used to tune the magnetization reversal of the Co/Ni bi-segmented nanowire. In Figure 7a, the parallel hysteresis loops measured at different temperatures point out the increase of both coercive field and remanence in Co segments. This effect has been associated in the literature to two main phenomena. First, the magnetostriction of Co and the different thermal expansion coefficients between the metallic nanowires and the ceramic alumina membrane, could induce mechanical stresses on the nanowires affecting their effective magnetic anisotropy [37,38]. On the other hand, the magnetocrystalline anisotropy of the hcp crystalline phase of Co is reduced with increasing temperatures. The Co nanowires fabricated for this work, whose details are shown in Section 2.1, present the hcp crystalline structure which its c-axis points perpendicular to the nanowire long axis. When increasing the temperature, the magnetocrystalline anisotropy of Co-hcp is reduced, the shape anisotropy becomes more dominant and thus an increase of both coercive field and remanence along the parallel direction to the nanowire axis is expected [39]. In terms of switching field, Ni segments show almost negligible temperature dependence in comparison with Co ones, resulting in an increase of the difference between the switching field of both segments as the temperature increases. Furthermore, with increasing temperatures, the two-step magnetization process should become more dominant while, for lower temperature, the magnetization reversal of coupled Ni/Co nanowires should be more homogeneous since their respective switching fields are closer to each other. 

**Figure 8:**
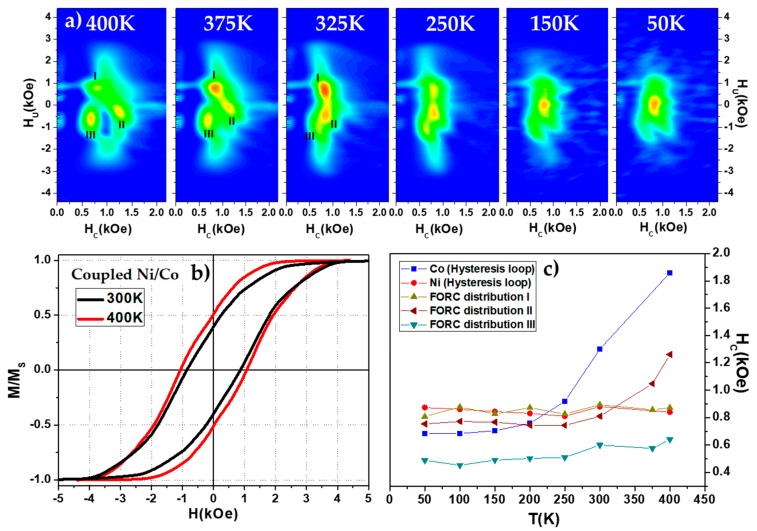
(**a**) Parallel FORC diagrams measured at different temperatures ranging from 400 K down to 50 K. (**b**) Parallel hysteresis loops of coupled Ni/Co bisegmented sample measured at 300 K (black) and 400 K (red). (**c**) Summarizes the coercive field of the Ni and Co hysteresis loops, and the main switching field of the different Ni/Co FORC distributions in the measured temperature range from 50 K to 400 K.

Figure 8 shows the proof of such switching field temperature dependence. At the highest temperature measured (400 K), a very complex FORC distribution is observed where three clear distributions can be seen (marked as I, II and III in Figure 8a), indicating a potential two-step magnetization reversal process. While the temperature is decreasing, the FORC diagram evolves towards a single distribution so that the coupled Ni/Co nanowires behave as a homogenous nanowire and the reversal of the magnetization is carried out in only one-step, i.e., a single and homogeneous FORC distribution. However, the coexistence of these two magnetization reversal behaviors is expected to occur over the whole measured temperature range. Considering only the parallel hysteresis loops of coupled Ni/Co bisegmented nanowire arrays shown in Figure 8b, this effect is hardly appreciable. However, taking a closer look, one can observe a more pronounced twist of the curve close to remanence at 400 K, thus indicating a more evident presence of a biphase contribution. In Figure 8c, the coercive field of the three FORC distributions can be tracked as a function of temperature. First, Distribution I shows an intrinsic switching field which is identical to the coercive field exhibited by the hysteresis loops of Ni segments. This distribution could reflect the switching of the Ni segment, as they would be isolated, however it would be unexpected that the Ni segments switching field is not modified with the coupling between Co segments. Moreover, as shown previously, strongly coupled segments would switch their magnetization in a single step at a switching field dominated by the magnetically softer segment, suggesting that Distribution I corresponds to the magnetization reversal of the whole bi-segmented Ni/Co nanowire driven by Ni segment. Furthermore, by decreasing the reversal field, the FORC diagram shows two different distributions, i.e., a reversal magnetization carried out in two steps (Distributions II and III). When the reversal field is further decreased, some Ni segments reverse their magnetization staying in an antiparallel configuration compared to their respective Co segments. If at this point the magnetic field is increased back to saturation magnetization, the portion of Ni segments that are aligned antiparallel, will switch back before its intrinsic switching field would be reached, due to the coupling with the Co segments at the interface (Distribution III) that forces the Ni/Co segments to be magnetized in parallel. This effect will show an effective switching field of such Ni segments lower than their intrinsic one due to that interface coupling. This behavior is also visible in the magnetization reversal of Co segments, which are represented by the Distribution II in the FORC diagrams of Figure 8a. The switching field at high temperatures ascribed to the Distribution II is noticeably higher than the one shown by Ni or Distribution I, but still lower than the coercive field of Co nanowire arrays, indicating that the Co segments (as the Ni ones) tend to leave the antiparallel configuration at lower switching fields. As the temperature is decreased, Distributions I and II collapse at the same coercive field, although around room temperature both distributions can still be clearly differentiated in the FORC diagram. Further decrease of the temperature results in the disappearing of both previous Distributions I and II, being substituted for a single one, indicating a single step reversal of the magnetization. In addition, at 400 K the relative intensity of Distributions II and III is higher than the intensity of Distribution I, indicating that the magnetization reversal of the bi-segmented nanowires is mainly carried out in a two-step process, while decreasing the temperature the intensity of the Distribution III decreases while the corresponding one of Distribution I increases accordingly. This demonstrates once again that reducing the temperature, the amount of bi-segmented Ni/Co nanowires that reverse their magnetization in a single step increase.

**Figure 9:**
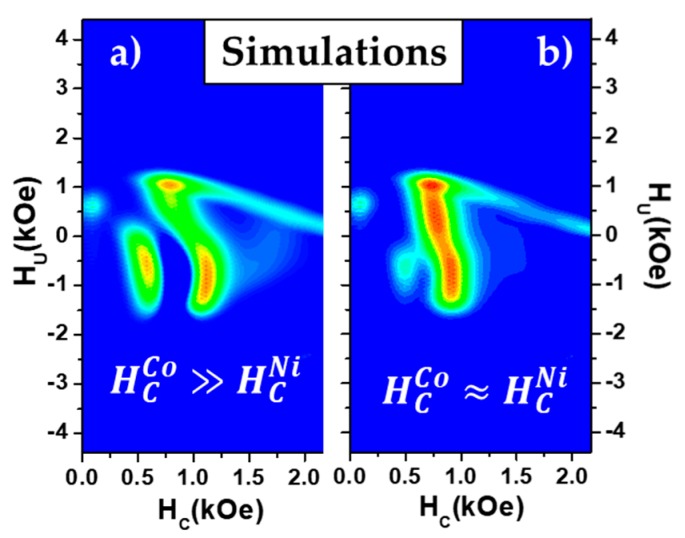
Simulated FORC diagrams of a coupled Ni/Co bisegmented nanowire array for the cases where the switching field of Co is much greater a) or slightly larger b) than the Ni switching field.

Finally, to validate the interpretation of the experimental results, the phenomenological contribution of a two-step magnetization process was implemented and simulation results are shown in Figure 9. For that purpose, two main contributions were considered: a homogeneous nanowire behavior and a two-step magnetization reversal contribution. In the first case, a switching field distribution whose main switching field is close to the Ni one ($H_{Ni/Co}^{homogeneous}=850\;Oe$) and an averaged mean field interaction value of α = −1400 Oe were fixed. In the case of the two-step magnetization process, the switching field of Co coupled segments was chosen as $H_{Co,A}^{coupled}=1100\;Oe$ and $H_{Co,B}^{coupled}=1000\;Oe$ for Simulations A and B (Figure 9a,b), respectively. In both cases, the coupled Ni segment was imposed to switch 100 Oe before the Co one when both are magnetized in parallel. In contrast, along the first order reversal curve starting from the antiparallel configuration, the Ni segment switches at $H_{Ni}^{FORC,coupled}=200\;Oe$ and $H_{Ni}^{FORC,coupled}=300\;Oe$ for Simulations A and B, respectively, leading in an effective switching field of 600 Oe in both cases. Although simulation results are in good agreement with the experimental data, providing the tools to detect two-step magnetization processes, the assumption of a mean field interaction model is an important limitation to extract quantitative information from such diagrams.

## 4. Conclusions

Ni/Co bi-segmented nanowire arrays were successfully fabricated and magnetically characterized. To extract the contribution of each nanowire composition to the magnetization reversal process as well as the different magnetic interactions between them, Ni and Co nanowire arrays, and both stacked nanowire arrays (decoupled Ni/Co sample), were also investigated. From hysteresis loops measured at room temperature, both Ni and Co nanowires present a well-defined easy magnetization axis parallel to the nanowire. Furthermore, Ni nanowires have been found to be magnetically softer than the Co ones, enabling the identification of each ferromagnetic segment contribution to the total magnetization when both types of nanowire segments are measured together. As a first result, the magnetic behavior of the decoupled Ni/Co sample corresponds to the superposition of the respective Ni and Co independent magnetization reversal behaviors. Moreover, when the nanowires are in contact at one end, hysteresis loops and thus the FORC distributions are modified drastically with respect to the not-interacting case, which suggests that some additional effects are provided by the magnetic coupling at the interface between Ni/Co segments. In a first approximation, the hysteresis loop of the coupled Ni/Co nanowire array suggests that the effective magnetization reversal process would be driven by the softer ferromagnetic phase, in this case Ni segment, as far as the overall magnetic hysteresis is reduced taking values closer to the Ni nanowire array. However, even from the hysteresis loop, two different magnetization reversal process can be distinguished. It results interesting the possibility to associate one of these processes with a magnetization reversal taking place in two steps and what would be the FORC signature of this kind of phenomenon. The FORC analysis made on this sample evidences the magnetic coupling between the Ni and Co segments, whose diagram has been associated with a system in which the antiparallel magnetization configuration in each bi-segmented nanowire is possible. Such antiparallel configuration as well as the magnetization reversal behavior associated to it depends strongly on the difference between the switching field of both nanowire segment compositions, finding two different FORC distributions with a critical switching field difference of around 500 Oe. As this difference increases, the probability to achieve the antiparallel configuration also increases, as observed in the temperature dependence of the FORC analysis in the bi-segmented Ni/Co nanowire array. The main result from this work lies in the FORC fingerprint identification characteristic of a two-step reversal magnetization of segmented nanowires. Such a result could shed light on the magnetic characterization and the understanding of vast nanostructure architectures that are, nowadays, under investigation.

## Figures and Tables

**Table 1:** Values of the coercive field, H_C_, extracted from the major hysteresis loop and mean switching field, MFI at saturation (α) and standard deviation (σ) of the SFD extracted from FORC for Co and Ni nanowire segment arrays.

Sample	$H_{C}^{\mathrm{HL}}\left(\mathrm{Oe}\right)$	$H_{\mathrm{SW}}^{\mathrm{FORC}}\left(\mathrm{Oe}\right)$	α(Oe)	σ(Oe)
Co	1296	1175	−2350	185
Ni	884	850	−600	135
